# Assessing the Impact of Efalizumab on Nail, Scalp and Palmoplantar Psoriasis and on Quality of Life: Results from a Multicentre, Open-label, Phase IIIb/IV Trial

**DOI:** 10.1111/j.1753-5174.2009.00023.x

**Published:** 2009-12

**Authors:** Andreas Katsambas, Ketty Peris, Gino Vena, Peter Freidmann, Gottfried Wozel, Esteban Daudén, Daiana Licu, Mauro Placchi, Michel De La Brassinne

**Affiliations:** * Department of Dermatology, Andreas Sygros Hospital, University of AthensAthens, Greece; † University of L'AquilaAquila, Italy; ‡ Dermatology Clinic, University of BariBari, Italy; § Southampton General HospitalSouthampton, UK; ¶ Department of Dermatology, Technical UniversityDresden, Germany; ** Hospital Universitario de la PrincesaMadrid, Spain; †† Merck Serono International S.A.Geneva, Switzerland; ‡‡ Department of Dermatology, Centre Hospitalier UniversitaireLiège, Belgium

**Keywords:** Efalizumab, Nail Psoriasis, Palmoplantar Psoriasis, Quality of Life, Scalp Psoriasis

## Abstract

This post-approval, open-label trial (*n* = 1266) assessed the efficacy of efalizumab, administered in accordance with the European label at that time, in patients with concomitant nail, scalp or palmoplantar psoriasis. Patients received subcutaneous efalizumab 1.0 mg/kg weekly for up to 20 weeks. By Week 12, an improvement from baseline of 50% or more was observed in 21.4% (181/844) of patients with nail psoriasis, 62.4% (718/1150) of patients with scalp psoriasis, and 51.4% (127/247) of patients with palmoplantar psoriasis. Quality of life improved throughout the trial, with a 50% median improvement in DLQI score after 12 weeks of treatment. Efalizumab showed promising efficacy in the treatment of nail, scalp and palmoplantar psoriasis, which was reflected in improvements in quality of life.

## Introduction

Psoriasis frequently affects the nails, scalp, palms of the hands and soles of the feet, which can have disabling consequences and a considerable negative impact on a patient's quality of life (QoL) [1–6]. It can be very difficult to treat; in particular, the nails and scalp are not amenable to many topical psoriasis treatments or phototherapy because the nail plate or hair, respectively, prevent adequate contact with the affected tissue [2,5–7].

Efalizumab is a recombinant monoclonal immunoglobulin G1 antibody that binds to the CD11a subunit of lymphocyte function-associated antigen type 1. Until early 2009 it was approved in Europe for the treatment of adults suffering from moderate-to-severe chronic plaque psoriasis. This study reports data from a large-scale, prospective post-approval trial, the first of its kind in psoriasis patients, which assessed the efficacy and safety of efalizumab therapy in patients treated according to the European label at that time. Separately reported findings from this trial indicated that efalizumab effectively controlled psoriasis in two-thirds of patients within 12 weeks, and that this was maintained in over three-quarters of patients who continued to receive treatment for a total of 20 weeks [8]. In this article we present results from this trial for patient subgroups presenting with nail, scalp or palmoplantar psoriasis, as well as QoL outcomes for the entire patient population.

## Methods

### Patients

The trial included patients aged ≥18 years with a diagnosis of moderate-to-severe plaque psoriasis who had failed to respond to, were contraindicated for, or were intolerant of other systemic therapies, including ciclosporin, methotrexate and psoralen plus ultraviolet (UV)A phototherapy. Key exclusion criteria included guttate, erythrodermic or pustular psoriasis as the sole or predominant form of psoriasis; withdrawal from previous efalizumab treatment as a result of lack of efficacy or an adverse event; history of opportunistic infections or ongoing uncontrolled infections or active tuberculosis (TB), or treatment for TB within 1 year prior to entry. Written informed consent was obtained from all patients enrolled in the trial.

### Trial Design

The trial design is described in detail elsewhere [8]. Briefly, after a single subcutaneous (s.c.) conditioning dose of efalizumab 0.7 mg/kg, eligible patients received open-label s.c. efalizumab 1.0 mg/kg once weekly for a further 11 weeks (first-treatment period). At Week 12, responders could opt to continue with efalizumab treatment for a further 8 weeks while nonresponders switched to an alternative approved anti-psoriasis medication for up to 12 weeks. Regardless of their response, patients could choose to discontinue anti-psoriasis medication at Week 12; these patients entered the observation period and were monitored without treatment for up to 8 weeks or until signs of worsening psoriasis were observed. Patients who responded during the first-treatment period but experienced worsening psoriasis during the observation period could then receive further treatment with weekly open-label s.c. efalizumab 1.0 mg/kg for a further 12 weeks. The trial was performed in accordance with the Declaration of Helsinki Guidelines for Good Clinical Practice, with approval by the independent ethics committee/institutional review board for each participating country.

### Assessments of Nail, Scalp and Palmoplantar Psoriasis

Nail Psoriasis Severity Index (NAPSI) assessments were made at baseline and at Week 12 of the trial. Psoriasis Scalp Severity Index (PSSI) assessments and Palmoplantar Pustulosis Psoriasis Area and Severity Index (PPPASI) assessments were made at baseline and at Weeks 4, 8 and 12. In each case, higher scores indicate greater disease severity.

### Assessment of Quality of Life

QoL was assessed at baseline and Weeks 2, 4, 8, 12 and 20 using the Dermatology Life Quality Index (DLQI) and the Medical Outcomes Study Short Form-36 (SF-36).

### Statistical Considerations

The intent-to-treat (ITT) population included all patients who received at least one dose of efalizumab and had at least one post-dose efficacy assessment.

Only subjects with baseline NAPSI, PSSI or PPPASI scores greater than zero were included in the nail, scalp or palmoplantar efficacy analyses, respectively. The proportions of patients achieving an improvement from baseline of ≥50% (NAPSI 50; PSSI 50; PPPASI 50) and ≥75% (NAPSI 75; PSSI 75; PPPASI 75) at Week 12 were determined.

Descriptive statistics were presented for SF-36 and DLQI data. For SF-36, a subscale score was included for patients who responded to at least half of the questions in that subscale; the mean score was calculated from the available responses.

For categorical data analyses, patients missing Week 12 data were considered to be nonresponders (worst-outcome imputation). For continuous data, patients with a missing value were excluded from the analysis at the time point for which the data were missing.

## Results

### Patients

Of the 1266 patients enrolled to the trial, 11 were excluded due to lack of post-baseline efficacy assessment data. Thus the ITT population comprised 1255 patients (68.5% male; mean age 46.2 years). Of these, 1084 completed the first 12 weeks of treatment, with 171 patients withdrawing from treatment for the following reasons: adverse event (*n* = 81); lost to follow-up (*n* = 8); protocol violation (*n* = 3); lack of efficacy (*n* = 52); other (*n* = 27). A total of 688 patients, most of whom had responded to treatment during the initial 12-week treatment period, continued to receive efalizumab for a further 8 weeks, for a total of 20 weeks of efalizumab therapy.

### Efficacy in Nail Psoriasis

**Figure 1:**
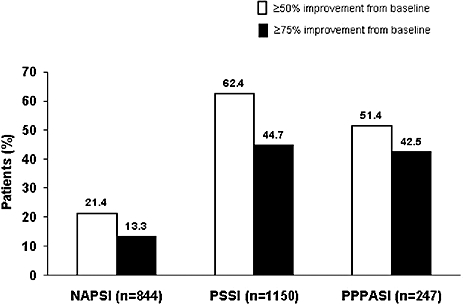
Proportions of patients achieving a 50% or 75% improvement from baseline to Week 12 in Nail Psoriasis Severity Index (NAPSI), Psoriasis Scalp Severity Index (PSSI), or Palmoplantar Pustulosis Psoriasis Area and Severity Index (PPPASI) score; intent-to-treat populations.

There were 844 patients with a NAPSI score greater than zero at baseline (Table 1). The median improvement in NAPSI score from baseline to Week 12 was 10% (interquartile range [IQR] 0–42.9). By Week 12, 21.4% (181/844) of patients achieved NAPSI 50, and 13.3% (112/844) achieved NAPSI 75 (Figure 1).

**Table 1:** Nail and Psoriasis Severity Index (NAPSI), Psoriasis Scalp Severity Index (PSSI) and Palmoplantar Pustulosis Psoriasis Area and Severity Index (PPPASI) scores at baseline and Week 12

	NAPSI	PSSI	PPPASI
Number of patients with score >0 at baseline	844	1150	247
Number (%) of patients with high score at baseline	353 (41.8)*	408 (35.5)†	109 (44.1)‡
Median score (range)			
At baseline	24.0 (1–80)	16.0 (1–72)	4.0 (0.2–43.2)
At Week 12	20.0 (0–80)	3.0 (0–60)	1.20 (0.0–53.0)

* Score ≥29;

† score >21;

‡ score ≥5.

### Efficacy in Scalp Psoriasis

A PSSI score greater than zero was recorded for 1150 patients at baseline (Table 1). By Week 12, there had been a median improvement in PSSI score of 73.3% (IQR 33.3–94.3) compared with baseline.

At Week 12, PSSI 50 and PSSI 75 responses were achieved by 62.4% (718/1150) and 44.7% (514/1150) of patients, respectively (Figure 1).

In many cases, a response to efalizumab was apparent early in treatment (Table 2), with over half of the patients classified as PSSI 50 responders at Week 12 having already achieved this response by Week 4 (*n* = 425).

**Table 2:** Median Psoriasis Scalp Severity Index (PSSI) and Palmoplantar Pustulosis Psoriasis Area and Severity Index (PPPASI) scores (interquartile range) by visit

	PSSI	PPPASI
Baseline	16.0 (6.0–28.0)	4.00 (1.20–11.20)
	*n* = 1150	*n* = 247
Week 4	9.0 (3.0–18.0)	2.40 (0.60–6.70)
	*n* = 1138	*n* = 242
Week 8	6.0 (2.0–12.0)	2.00 (0.20–5.60)
	*n* = 1093	*n* = 229
Week 12	3.0 (1.0–10.0)	1.20 (0.00–4.80)
	*n* = 1042	*n* = 216

### Efficacy in Palmoplantar Psoriasis

There were 247 patients with a PPPASI score greater than zero at baseline (Table 1).

At Week 12, the median improvement in PPPASI score from baseline was 69.4% (IQR 0.0–100.0), with 51.4% (127/247) of patients achieving PPPASI 50 response, and 42.5% (105/247) of patients achieving PPPASI 75 response (Figure 1).

### Quality of Life

#### Dermatology Life Quality Index

**Figure 2:**
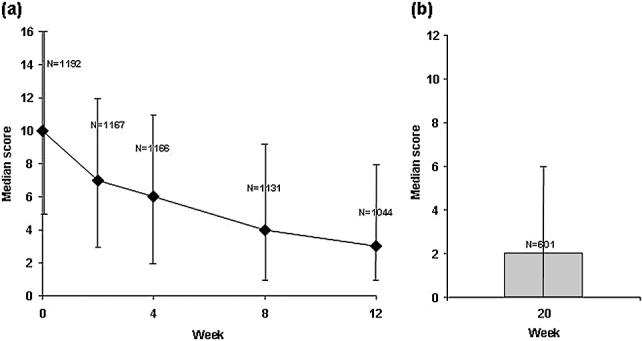
Median (interquartile range) Dermatology Life Quality Index score (a) Weeks 0–12, intent-to-treat population; (b) Week 20, continuous-treatment population.

A total of 1192 patients from the ITT population had DLQI data at baseline while 1044 patients had DLQI data at Week 12. DLQI scores ranged from 0 to 30 at baseline, with a median of 10 (IQR 5.0–16.0). Median DLQI scores improved steadily from baseline to Week 12 (Figure 2a). Among patients from the ITT population who had DLQI data at Week 12, the median improvement from baseline was 5 points (IQR 1.0–10.0).

A total of 601 patients from the ITT population continued efalizumab treatment beyond Week 12 with recorded DLQI data at Week 20. The median DLQI score for this subset of patients at Week 20 was 2.0 (IQR 0.0–6.0; range 0.0–30.0) (Figure 2b). This represents a median improvement from baseline of 60% (6.0 points; IQR 2.0–12.0).

#### Medical Outcomes Study Short Form-36

SF-36 scores ranged from 40.0 to 792.0 (median 571.5; IQR 419.0–665.3) at baseline and improved steadily from baseline to Week 12. At Week 12, 986 patients from the ITT population had SF-36 data recorded. Among these patients, the median SF-36 score at Week 12 was 645.0 (range 70.0–800.0; IQR 503.0–708.0). This represents a mean improvement from baseline of 58.3 points (standard deviation 144.5; IQR −10.8–129.4).

Among those patients who continued efalizumab treatment beyond Week 12 and had SF-36 data at Week 20 (*n* = 563), the median SF-36 score was 658.5 (range 68.0–800.0; IQR 551.5–716.5) at Week 20, which was a 9.4% improvement from baseline.

### Safety

Efalizumab was well tolerated. Full safety data are reported in the primary trial publication [8].

## Discussion

This report describes the results arising from the secondary analysis of a large-scale prospective post-approval trial examining the efficacy and safety of efalizumab in patients with moderate-to-severe, chronic plaque psoriasis who had failed to respond to, had contraindications for, or were intolerant of other systemic therapies, including ciclosporin, methotrexate and psoralen plus UVA phototherapy. Evaluation of the primary endpoints of this trial have shown the safety and efficacy of efalizumab across the patient population as a whole [8].

A subgroup analysis of patients presenting with nail, scalp or palmoplantar psoriasis within this same trial are described herein. The data presented indicate that treatment with efalizumab for 12 weeks also resulted in a marked improvement in these conditions. In patients with scalp or palmoplantar involvement, over 40% of patients achieved at least a 75% improvement in symptoms by Week 12. Similarly, of the patients with nail involvement, more than one-fifth achieved at least a 50% improvement in symptoms by Week 12. These results support and extend the favourable findings of earlier smaller-scale trials reported in the Latin American CONTROL I trial [9] or the placebo-controlled Phase IV hand and foot plaque psoriasis trial [10].

Psoriasis can greatly influence a patient's self-esteem and sense of well-being, and consequently is detrimental to their QoL. The beneficial effects of efalizumab in the patients enrolled in this trial were paralleled by steady improvements in QoL throughout the initial 12-week efalizumab treatment period. This trend, along with the 50% (5-point) improvement in DLQI score from baseline to Week 12 of efalizumab treatment, is also in accordance with the results of previous studies examining QoL following treatment in psoriasis patients [11–15].

In light of post-marketing surveillance of patients with psoriasis receiving efalizumab continuously for more than 3 years, in which opportunistic infections were reported, and in particular cases of JC virus infection resulting in progressive multifocal leucoencephalopathy (PML), the European Medicines Agency evaluated all safety data. It concluded that the benefits of efalizumab treatment no longer outweighed the risks associated with the drug and recommended suspension of marketing authorization on 19 February 2009.

This trial has confirmed that efalizumab, administered in accordance with the European label available at that time, provided effective control of moderate-to-severe plaque psoriasis. This latest analysis indicates that efalizumab can also be an effective treatment in difficult-to-treat forms of the disease, including nail, scalp or palmoplantar psoriasis, with profound effects on a patient's QoL.
